# Are Women More Vulnerable to Flooding Than Men in an Aging Japanese Society?

**DOI:** 10.3390/ijerph20021299

**Published:** 2023-01-11

**Authors:** Juan Fan, Guangwei Huang

**Affiliations:** Graduate School of Global Environmental Studies, Sophia University, Tokyo 102-8554, Japan

**Keywords:** flood vulnerability, aging society, gender, physical ability, evacuation

## Abstract

It is a well-accepted notion that women are more vulnerable to natural disasters than men, especially in developing countries. However, in developed countries, how women’s empowerment by economic and social development has reduced the gender gap in vulnerability remains insufficiently answered. As Japan passed its golden age, moving into an aging society, a study on how the gender difference in flood vulnerability has evolved can contribute to a better understanding of the types and causes of vulnerability, leading to better flood risk management in a new social context. Following this thinking, the present study conducted a longitudinal analysis using representative flooding cases in Japan over a period of forty years. It found that the women’s fatality rate increased with age much faster than men’s in the 1980s but reversed in a recent major flood disaster. It also revealed that most flood disaster victims were elderly in recent years. These findings suggest that the flood vulnerability at present is more driven by age-related physical ability decline, much less relevant to gender. Based on the results, it proposed a new framework for assessing flood vulnerability in an aging society. Such outcomes can help with the better formulation of flood management policies and probing into solutions.

## 1. Introduction

Flood risk management has been studied intensively and extensively and has been gaining more attention as climate change would increase the frequency of flooding [1] and cause more severe consequences [2]. A recent study indicated that the risk of catastrophic megafloods in California has doubled due to global warming, although California largely struggles with severe drought [3]. Another publication showed that if it were not for the impact of climate change, up to 50 percent of Houston’s Harris County residences would not have been flooded by Hurricane Harvey in 2017 [4]. For the western U.S., it projected that flood damage could increase three times compared to 2019 under a particular climate change scenario.

In Japan, climate changes are causing more frequent and extreme rainfall events, increasing human losses and economic damage in recent years. Typhoon Hagibis, which struck in mid-October 2019, caused ¥1.86 trillion of damage, the highest on record for a single event. Typhoon Hagibis resulted in record rainfall in a wide-ranging area, breaking 142 levees nationwide. The disaster resulted in 87 dead and damage to over 80,000 buildings [5].

In 2018, Typhoon Jebi claimed 14 lives, injured 934, and damaged more than 50,000 houses [6]. It also flooded a runway and Terminal 1 of Kansai International Airport [7], which serves 80,000 passengers a day. Besides, some 3000 passengers were stranded overnight when a tanker crashed into the only bridge that links the airport to the mainland. 

Considering such a flood disaster trend, a research question to address for better flood risk management is who is the most vulnerable to the increase of flood hazards and why. Certain factors make specific groups structurally and systematically more vulnerable such as gender, age, disability, income, and education. Notably, women are often considered the most vulnerable in times of disaster because they are exposed to more dangers due to their various roles, such as taking care of children, older family members, and people with disabilities before, during, and after a disaster event. At the same time, men tend to respond to natural disasters differently. As Hurricane Katrina passed New Orleans, about 80 percent of the people left in New Orleans after the mandatory evacuation was issued were women, although women accounted for only 54 percent of the city’s population.

Cyclone Gorky, which hit Bangladesh in 1991, caused around 140,000 deaths. The disparity between genders in terms of survival from this event was approximately 14:1. In other words, this cyclone killed 14 women for every man [8]. Similarly, in the 2004 Indian Ocean tsunami, 70 percent of the 250,000 fatalities were women [9].

On the other hand, a study using gender-disaggregated data from disasters between 1981 and 2002 in 141 countries showed that natural disasters lower women’s life expectancy more than men’s. Further, the higher the women’s socioeconomic status, the weaker this effect on the gender gap in life expectancy [10]. It is the first systematic and quantitative analysis of gender differences in natural disaster mortality.

**Figure 1:**
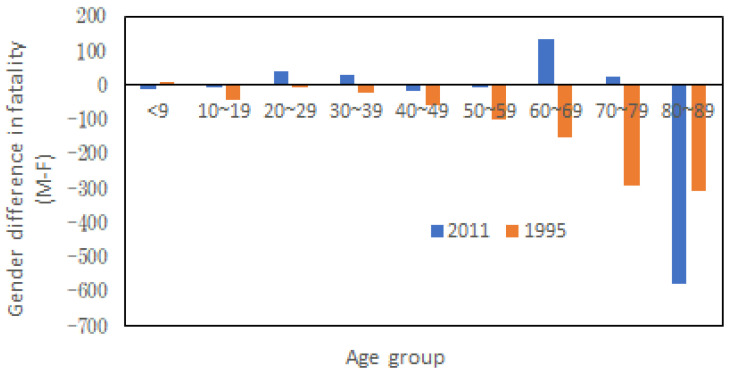
Gender gap in victims (men-women) in different age groups during the Hanshin-Awaji Great Earthquake and the Great East Japan Earthquake (Data source: e-Stat, Japan).

It was well reported that more women died than men in both Hanshin-Awaji Great Earthquake in 1995 and the Great East Japan Earthquake in 2011. Nevertheless, a further look into the age distribution of victims in the two cases indicates that more women died than men in almost all age groups in 1995. But only more aged women perished than men in 2011, as shown in Figure 1. In 2011, 92.4% of deaths were due to Tsunami-caused drowning, and in 1995, 83.3% were caused by house collapses. Given these facts, one may tend to hypothesize that aged female is the most vulnerable to water-related disasters in an aging society. Validate or rejecting is an important step forward for better disaster policy making.

Another study of flood fatality in eight European countries for the period of 1980–2018) also revealed that the gender difference in flood fatality is age-dependent [11]. Males were more numerous than females in the age group of 30–49, while females were more numerous than males in the age class above 65. 

The probability of life lost during a flood depends on many factors related to an individual’s physical ability, community strength, and societal system. To further reduce flood fatality, more disaggregated analyses are needed to understand better what caused life loss during a flooding event. 

The present study aimed to explore the gender difference in flood fatality in relation to age structure. It used several flooding cases in Japan at different times to serve as longitudinal profiling of the gender of victims. Furthermore, it proposed a new framework to assess flood vulnerability in an aging society. The value of this work lies in clarifying a sensitive and vital disaster-related social issue, which is often misperceived. 

## 2. Materials and Methods

This study employed a case study approach because it can provide an in-depth, multi-faceted understanding of a complex issue in its real-life context. It may be characterized as a “naturalistic” research design in contrast to an “experimental” design because a case study is both the process of learning about the case and the product of the learning, as stated by Stake [12]. Furthermore, the case study approach is often an inherently mixed method if it uses more than one form of data within a research paradigm or more than one form from different paradigms. The present study used qualitative and quantitative data to explore the question, “Have more women died than men in flood disasters?”. Besides, the research design lends itself well to explaining causal links. As a result, a theoretical framework was proposed based on analyses for assessing flood vulnerability in an era of aging. 

### 2.1. Case Selection

**Figure 2:**
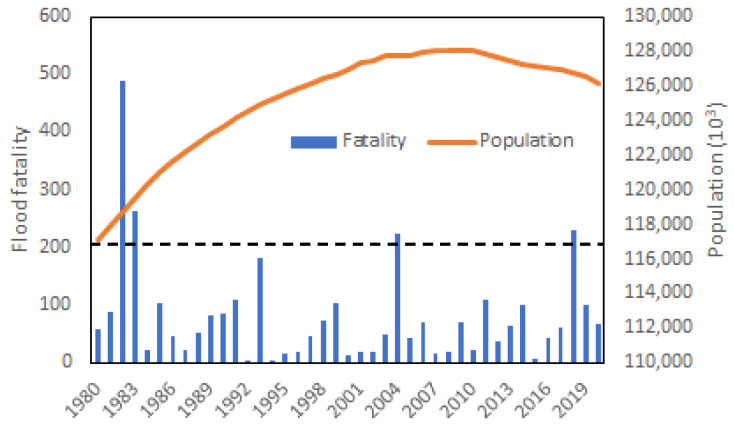
Flood fatality in Japan for 1980–2020 (Data source: e-Stat, Japan).

Figure 2 shows Japan’s number of flood disaster victims and population from 1980 to 2020. Over the past four decades, there were four years when the annual flood fatality exceeded 200. They were in 1982, 1983, 2004, and 2018. Since flood fatality data for 1999–2017 had been studied by Ushiyama [13], the present study targeted 1982, 2018, and 2020 and focused on the major flood event in each selected year. The case selection was justifiable since the 2018 and 2020 floodings were the recent major flood disasters in Japan, reflecting the current situation. The data on victims’ gender and age were collected and analyzed from various view angles. 

#### 2.1.1. 1982 Case

On 23 July 1982, heavy rainfall having the intensity of 100 to 187 mm per hour killed 299 people and caused massive damage to Nagasaki City and its vicinity [14]. More than 1500 homes were destroyed or seriously damaged, and about 18,000 homes were flooded. The total loss was as high as ¥315.3 billion. Although this old case had been studied extensively, the present study used this case to shed new light on women’s vulnerability and for comparison. 

#### 2.1.2. 2018 Case

From late June to early July 2018, successive heavy rainfalls in southwestern Japan resulted in widespread flooding. Okayama, Hiroshima, and Ehime prefectures were the worst affected regions. The fatalities were 231, more than 17,000 homes were destroyed or damaged, and 3800 homes flooded. The economic losses in Okayama, Hiroshima, and Ehime prefectures were 402.8 billion, 344.7 billion, and 125.7 billion, respectively [15,16]. 

#### 2.1.3. 2020 Case

The year 2020 was chosen because it can highlight the vulnerability of the aging society to flood disasters, although the number of victims in 2020 was less than 100. Besides, the Kuma River flooding caused an inundation depth of 4.3 m in one area of Hitoyoshi City, making a new flooding depth record in the region since 1965 [17,18,19].

## 3. Analysis and Results

### 3.1. For 1982

**Figure 3:**
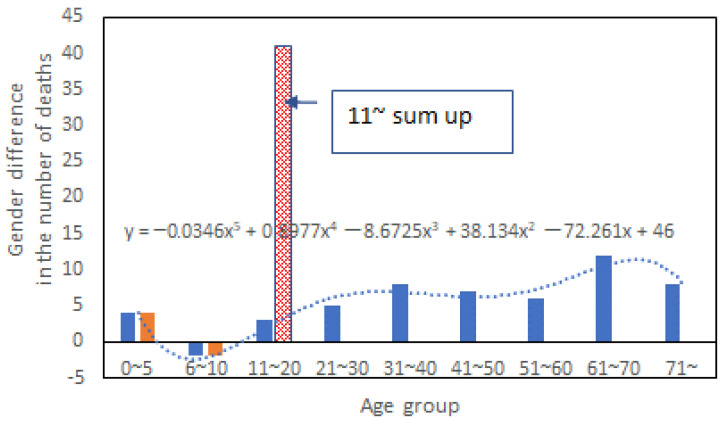
In-house death difference between men and women for different age groups.

**Figure 4:**
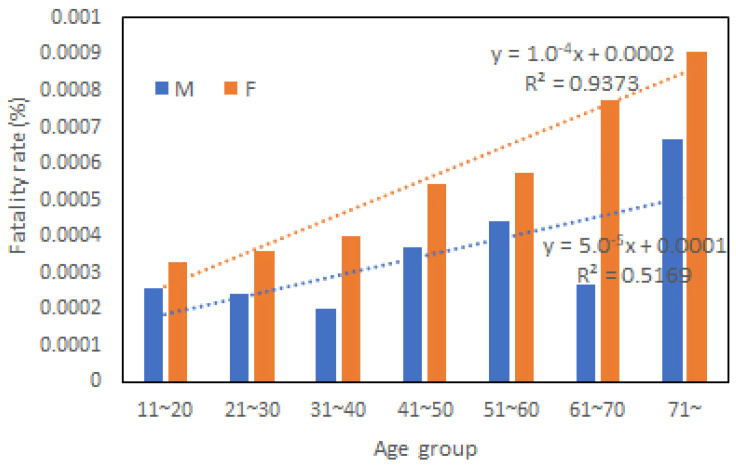
Relationships between age, gender, and fatality rates.

In the Nagasaki disaster in 1982, 87.6% of the deaths were caused by rainfall-induced landslides, and most of them were buried alive [14]. For those in-house deaths, the age and gender information were compiled by Matsuda [20], and the gender gap in the death toll was shown in Figure 3. This record indicated that female mortality was 1.5 times higher than that of men in this disaster, as concluded by Matsuda. By summing up all the gender differences in death toll (the number of male victims minus the number of female victims) for age groups above 10, one can see clearly that age 10 was a dividing line for the gender gap in this case. Besides, the gender difference variation across ages fitted a polynomial of degree 5, as shown in Figure 3. Mathematically, a quintic polynomial has one to three inflection points, which implies a low possibility of having a general trend. To further examine the relationship between age, gender, and fatality, we calculated the fatality rate (death toll/population of the affected administrative area) for different age groups and genders using Nagasaki City’s population statistics data for 1985. The results are shown in Figure 4. For age groups above 10, a strong linear relationship between age and death was observed for females (the orange dotted line) but not males (the blue dotted line). Above that, the fatality rate increased with age much faster for women than men. This finding provided new evidence 40 years after the disaster that women were more vulnerable than men in the early 1980s. However, the cause of this gender difference was left unexplained. According to the Cabinet Office of Japan, the evacuation rate for those who received evacuation calls was 27.3% in this disaster. How the gender gap in the fatality rate was related to evacuation behavior and other factors deserves in-depth exploration. 

**Figure 5:**
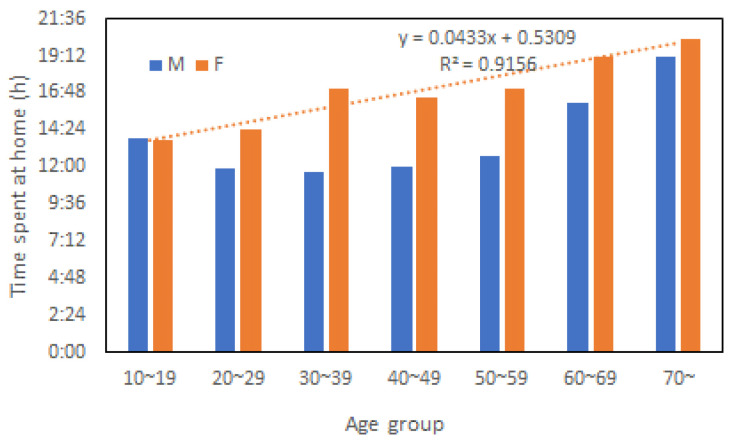
Gender difference in time spent at home for different age groups.

Figure 5 shows the survey results by NHK in the 1990s on time spent at home by different ages and genders [21]. On average, women spend 20% more time at home than men and the time spent at home increased linearly with age as indicated by the dotted line in the figure. Therefore, the time spent at home could be considered a factor behind the linearity of women’s fatality with age. So far, such a view angle has not been taken into flood risk analysis. A survey in two districts of Nagasaki City after the disaster showed that 8.3% of males were reluctant to evacuate, while the figure was 1.3% for females [22]. However, as Japan was a male-dominated society in the 1980s, If a family’s husband decided not to evacuate, the whole family would not evacuate. On the other hand, the husband may evacuate senior and very young family members first and decide about his own evacuation later. No matter what may happen, the decision-making process within a family could affect the evacuation rate. So, it is an essential issue for better flood risk management and needs to be addressed. 

### 3.2. For 2018

**Figure 6:**
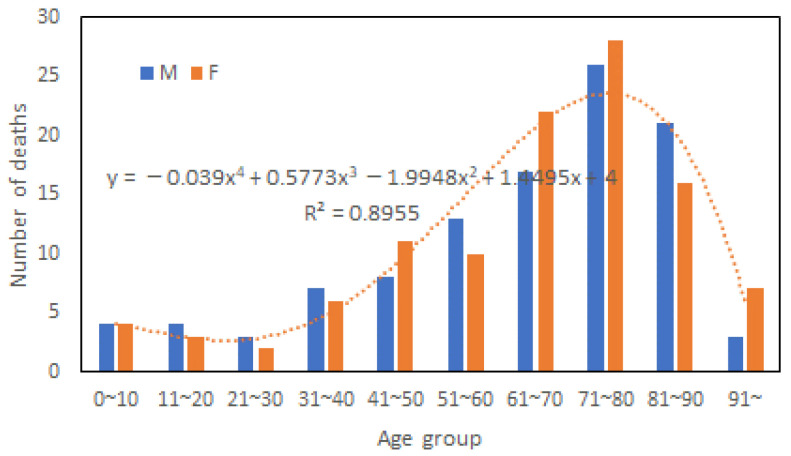
Death tolls in different age groups in 2018 (Data source: e-Stat, Japan).

Figure 6 shows the number of male and female victims in different age groups in the 2018 flood disaster, obtained from e-Stat, a portal site for Japanese Government Statistics. The average ages of victims were 62.6 and 64.1 for males and females, respectively. Then, Mann-Whitney U-Test was performed to check if the difference in average death age between male and female groups is statistically significant. It was found that the z-score is 0.23682 and the *p*-value is 0.81034, indicating the difference is not significant at *p* < 0.05. Comparing the age distribution curves of the 1982 and 2018 victims highlighted that the proportion of elderly victims was much higher in 2018 than in 1982. It can also be observed that the victim’s age distribution curves can be approximated by a polynomial of degree 4, as shown in Figure 6. Consequently, the variation of gender difference in fatality with age did not fit a polynomial.

**Figure 7:**
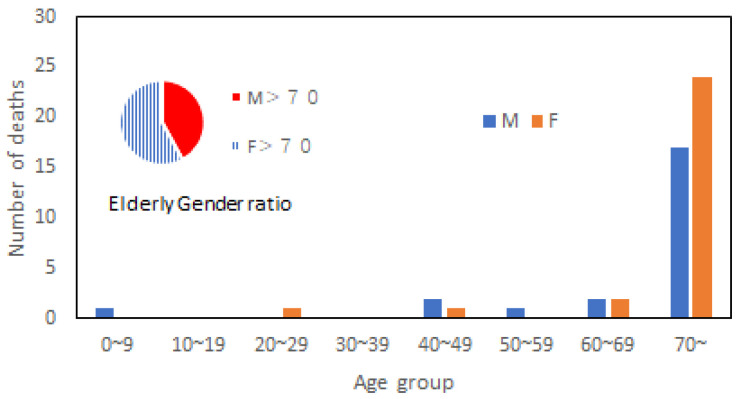
Death tolls in different age groups and elderly gender ratio in Mabi Town.

Similar to 1982, rainfall-induced landslides occurred in many places, resulting in 119 deaths. In Hiroshima Prefecture, the percentage of landslide-related deaths was as high as 90%. In Mabi Town, the hardest hit area in Okayama Prefecture, 51 people perished, and most died of drowning in their houses. Based on the data from Asahi News Digital [23], Figure 7 presents the victim’s age distribution curve. Among senior victims, 42% were men, and 58% were women. From this data, one may tend to conclude that senior women were more vulnerable than senior men. Nevertheless, the population pyramid for this region is that males above 70 account for 42%, while females above 70 account for 58%. The elderly victim’s gender ratio is the same as the overall senior gender ratio. Therefore, the statement that females are more vulnerable than males cannot be justified, although the absolute number of victims was higher for females than males. Besides, the number of senior victims appeared to increase exponentially from 40. 

Indeed, most of the victims were older people with disabilities who needed support for evacuation. However, a study reported that 90% of Mabi Town victims lived alone or with just one family member [24]. Family structure is probably a more important risk factor than gender in an aging society.

### 3.3. For 2020

**Figure 8:**
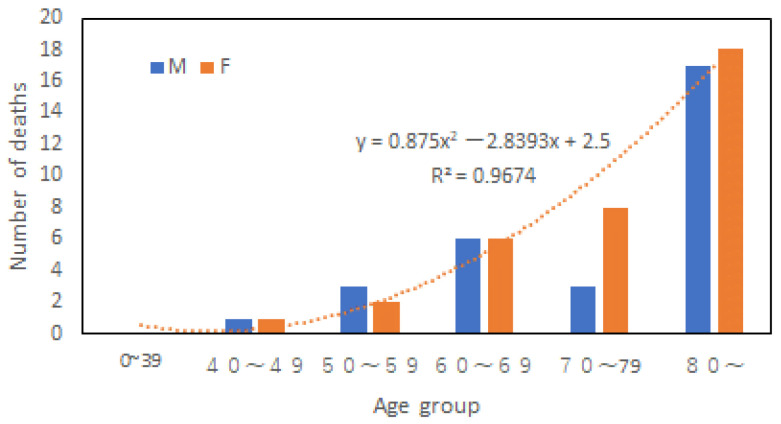
Death tolls in different age groups in the Kuma River flooding (Data source: e-Stat, Japan).

Figure 8 shows the number of male and female victims in different age groups in the Kuma River flooding disaster in 2020. A significant difference between this disaster and the disasters in 1982 and 2018 is that there were no young victims, and this may indicate that families with children and young people are now better prepared for flood disasters. Among all victims, males and females accounted for 46% and 54%, respectively. According to the population data of Hitoyoshi City, males and females above 40 at present are 44% and 56%, respectively. Among victims above 70, 43% were men, while 57% were women. However, males and females above 70 in the city at present are 39% and 61%, respectively. Therefore, it suggests that aged male is not less vulnerable than old female. Besides, the increase of victims with age fits into a quadratic polynomial.

**Figure 9:**
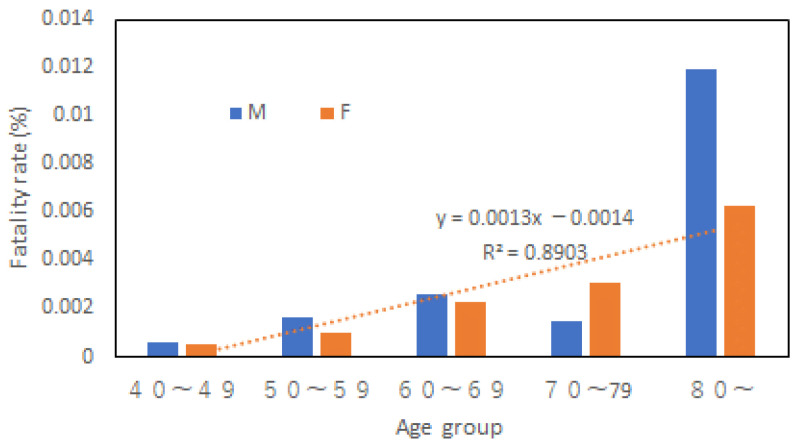
Fatality rates in different age groups and genders.

Figure 9 shows the fatality rates for both genders in this disaster. Like the 1982 case, women’s fatality rate increased linearly with age. But men’s fatality rate did not follow a linear law. It increased much faster with age than women, which is second evidence that women may not be the most vulnerable in an aging society of the developed world.

Regarding the cause of death, 81% of male victims died of drowning, while the figure was 83% for females. Furthermore, the death toll resulting from in-house drowning were 17 for female and 15 for male. Among them, nine women and five men lost their lives in a nursing home for the elderly close to the junction between the Kuma River and a tributary, where a levee breach occurred. At the time of the flooding, only five staff took care of 60 senior residents. The limited workforce could not move all to the second floor before the flood waters broke into the facility. Consequently, 14 people on wheels were left on the first floor and perished in flooding [19]. This tragedy suggests that physical disability or aging-induced decline in physical functions could be a more important factor than gender in analyzing flood fatality, especially for senior victims. Nevertheless, this factor has been very much understudied. 

According to studies in the field of geriatrics, the balance power of a person in the 60s and 70s is 30% and 20% of that in the 20s, respectively. In general, the worsening of physical function was accelerated in terms of the rate of change among women and those in older age groups [25,26]. However, as far as flood-induced fatality is concerned, impairment in balance performance can be considered more relevant since it is a commonly accepted risk factor for falling in older people. A study of balance performance in people at retirement age showed that when the data were normalized for height, no differences were found in static balance performance between men and women [27]. Therefore, flood emergency management should target senior men and women equally.

### 3.4. Framework Development

Over the past several decades, vulnerability has become a widely used analytic tool in various research fields, from disaster management, climate change, and food security to poverty reduction, although without a unanimously agreed definition of the term. Due to different conceptualizations and interpretations of vulnerability in various fields, there needs to have more clarity regarding the notion of vulnerability. To integrate different views on vulnerability and transform the relative concept into a more universal and usable tool, a step forward is to formalize it using mathematical expression [28]. 

A formulation of vulnerability was proposed by the Disaster Reduction Institute [29], in which vulnerability is seen as a combination of Exposure, Susceptibility, and Coping Capacity. The mathematical notion is given below:

(1) $$\mathrm{Vulnerabilty}=\frac{\mathrm{Exposure}\;\times \;\mathrm{Susceptibility}}{\mathrm{Coping}\;\mathrm{Capacity}}$$

where exposure is defined as the degree, duration, and extent to which a system is in contact with, or subject to, perturbation. Susceptibility refers to the factors and attributes that make a community or society more or less likely to be negatively affected by perturbation. Coping capacity is defined as the ability to cope with, absorb, and adapt to hazard impacts. It is the product of planned preparation before a disaster, emergency response during the disaster, and post-disaster reconstruction.

Exposure can be characterized by the total population living in the 1 in 100-year floodplain [30,31] or estimated according to potential property damage [32]. It depends on meteorological conditions and population distribution. A flood hazard map combined with population and housing data can provide a good quantification of flood exposure.

Susceptibility is a function of various parameters such as surface elevation, slope, distance to a river, soil type, and income, to name a few [33]. A work by Goumrasa et al. [33] provided a flood susceptibility assessment using GIS and AHP methods. Another study by Wang et al. [34] presented a flood susceptibility mapping framework involving an adaptive neuro-fuzzy inference system with two metaheuristic methods of biogeography-based optimization and an imperialistic competitive algorithm.

Since coping capacity consists of internal and external components, its assessment should encompass household conditions, community conditions, early warning system performance, emergency response capability, and post-disaster recovery management. Household conditions, an internal part of the coping capacity, should include family structure, income status, education level, and disaster preparedness. Community conditions, early warning system performance, emergency response capability, and an external part of the coping capacity, should include emergency drills and exercises at the community level, emergency shelter and evacuation route planning, emergency information delivery systems, and rescue operation capabilities. Post-disaster recovery management is internal and external, requiring individual efforts and public support in various ways. 

Wang et al. [35] developed a model to assess the coping capacity for Typhoon-caused disasters. However, the 17 indices used to evaluate typhoon coping capacity were mainly external. The development of coping capacity assessment methods remains a big challenge. 

In an aging society, seniors’ susceptibility increases as their physical strength declines. In Hitoyoshi City, we interviewed 23 senior residents. Of the question of whether or not they heard the emergency broadcast, 30% replied not. 

According to a study [36], hearing sensitivity declines more than twice as fast in men as in women at most ages. Women have more sensitive hearing than men at frequencies above 1000 Hz, but men have more sensitive hearing than women at lower frequencies. In the age groups of 60–69 and 70–79, the hearing alterations became steeper in females than in males. As a result, the gender differences in hearing smoothed significantly.

In an aging society, building a mutual assistance community for disaster management is essential for saving lives. Nevertheless, strategies for promoting such a system are limited, and the methodology for taking this aspect into coping capacity assessment has not been pursued up to now. Besides, according to a survey by the Ministry of Health, Labor and Welfare, Japan, the average annual income for the elderly is about 49% of other households [37]. The economic coping capacity of the elderly for post-disaster reconstruction is another issue deserving further study.

**Figure 10:**
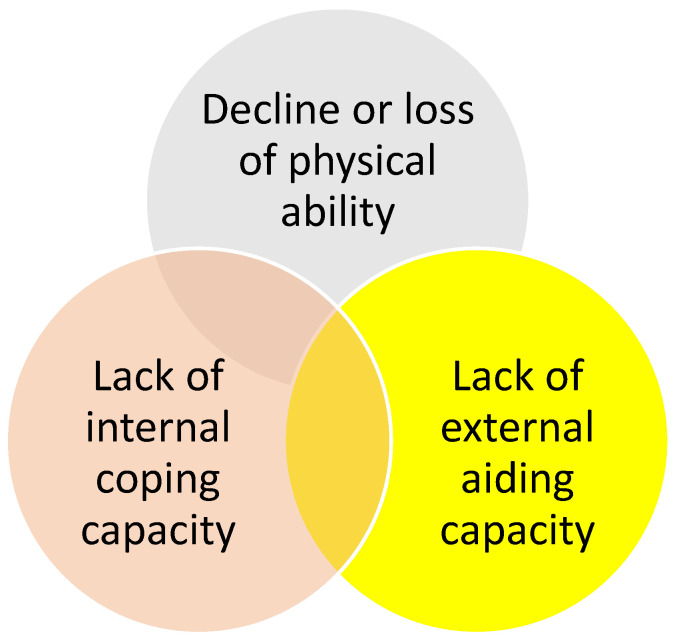
Framework for assessing flood vulnerability in an aging society.

Based on these facts, a new framework for assessing flood vulnerability in an aging society is presented in Figure 10. It consists of three pillars. The first pillar is the decline or loss of physical abilities such as walking and hearing. The second is the need for internal coping capacities, which depend on family structure. Elderlies living alone or with elderly spouses are often not capable of evacuating by themselves. The third is the need for external aiding capacity. A good combination of self-help, mutual aid, and public assistance is critical to minimize disaster damage. However, in an aging society, young people could be scarce in many communities. Consequently, how to build up a neighborhood-based mutual aid for emergency response and recovery is a mission to pursue. 

The Third Assessment Report of the Intergovernmental Panel on Climate Change (IPCC) describes vulnerability as a function of the character, magnitude, and rate of climate variation to which a system is exposed, its sensitivity, and its adaptive capacity [38]. Compared to the IPCC definition, the proposed framework is highly operational and tailor-made for the Japanese aging society. For example, it may guide local communities to secure more staff for emergency response and promote better evacuation plans for those with significant physical ability decline. 

## 4. Conclusions

The present work contributed to a better understanding of flood vulnerability concerning gender via a longitudinal study. The main findings are summarized below.

- In the 1980s, women were more vulnerable to water-related disasters than men, and the female fatality rate increased with age linearly and faster than men.
- Although the absolute number of water-related disaster victims in Japan in recent years was still higher for females than males, it is not evidence of women’s vulnerability but a reflection of the fact that there are more old women nowadays in Japan.
- Today, flood vulnerability in Japan is more driven by aging than gender. Such a change in flood vulnerability from gender-related to gender-neutral has not been reported up to now.
- The fatality rate in a recent flood disaster increased with age much faster for males than females.
- Moreover, the number of victims increased with age quadratically or exponentially in recent years.
- Finally, a new framework was proposed for assessing flood vulnerability in an aging society.

Knowing the potential risk associated with different age groups and genders in a local context is essential for preparing a better community-based emergency response plan. So, the present study’s outcomes enrich the vulnerability study literature and serve as a call for further work toward effective flood emergency management. Furthermore, the proposed new framework can help with better policymaking and exploring solutions.

## Data Availability

Available upon request.
